# Comparison of *Plasmodium falciparum *allelic frequency distribution in different endemic settings by high-resolution genotyping

**DOI:** 10.1186/1475-2875-8-250

**Published:** 2009-10-30

**Authors:** Sonja Schoepflin, Francesca Valsangiacomo, Enmoore Lin, Benson Kiniboro, Ivo Mueller, Ingrid Felger

**Affiliations:** 1 Swiss Tropical Institute, Socinstr 57, 4002-Basel, Switzerland; 2 Papua New Guinea Institute of Medical Research, PO Box 60, Goroka, Eastern Highland Province 441, Papua New Guinea

## Abstract

**Background:**

The diversity of genotyping markers of *Plasmodium falciparum *depends on transmission intensity. It has been reported that the diversity of the merozoite surface protein 2 (*msp2*) is greater in areas of high compared to low endemicity, however, results for *msp1 *were inconsistent. These previous reports relied on low resolution genotyping techniques.

**Methods:**

In the present study, a high-resolution capillary electrophoresis-based technique was applied to genotype samples from areas of different endemicity in Papua New Guinea and Tanzania. For both endemic settings, the diversity of *msp1 *and *msp2 *was investigated; the mean multiplicity of infection (MOI) and the F_ST _values were determined to investigate whether more accurate sizing generates different results.

**Results and Conclusion:**

The results of the present study confirmed previous reports of a higher mean MOI for both marker genes and increased genetic diversity in areas of higher endemicity as estimated by the total number of distinct alleles for *msp2*. For *msp1 *a minor increase in diversity was observed. Measures of between population variance in allele frequencies (F_ST_) indicated little genetic differentiation for both marker genes between the two populations from different endemic settings. MOI adjusted for the probability of multiple infections sharing the same allele was estimated by using the *msp2 *allele frequency distribution and the distribution of observed numbers of concurrent infections. For the high-resolution typing technique applied in this study, this adjustment made little difference to the estimated mean MOI compared to the observed mean MOI.

## Background

*Plasmodium falciparum *populations are highly diverse and individual hosts are often simultaneously infected by multiple parasite clones. In order to discriminate parasite clones within one host PCR-based genotyping of a number of different marker genes has been established. Among these are the merozoite surface proteins 1 and 2 (*msp1 *and *msp2*) and the glutamate-rich protein (*glurp*), which show extensive length polymorphism and can, therefore, be distinguished by electrophoresis on agarose or polyacrylamide gels.

Recently, capillary electrophoresis (CE) based genotyping of *msp2 *was shown to have a much higher discriminatory power than previously applied techniques [1]. This technique has been proven useful in areas of high endemicity, where individuals are simultaneously infected with many different parasite clones. It has also been useful for distinguishing recrudescent from new infections after anti-malarial treatment [2] and in tracking individual parasite clones over time in longitudinal surveys, where consecutive blood samples were collected from each individual [1]. Similar to the CE based *msp2 *genotyping, a CE protocol for genotyping the polymorphic block 2 of the *msp1*gene was applied in the present study.

The focus of this study was to assess the impact of transmission intensity on the genetic diversity of the two marker genes *msp1 *and *msp2*. Most previous studies have shown a greater diversity for *msp2 *in high endemic settings [3,4], but for *msp1 *conflicting results were reported [5-12].

These data all relied on low-resolution genotyping consisting of fragment sizing on agarose gels with individual genotypes defined as bins of ≥ 20 base pairs. Using this approach several distinct fragments may be combined within one genotype bin. Greenhouse *et al *[13] studied the impact of transmission intensity on the accuracy of genotyping and found that with increasing transmission intensity and, therefore, increasing complexity of infection, genotyping becomes less accurate. These findings are not only important for recrudescent-reinfection typing in anti-malarial drug trials, but also for assessing the impact of transmission intensity on genetic diversity. The authors concluded that a genotyping technique with higher discriminatory power is needed for genotyping samples from areas of high transmission intensity. In the study presented here an improved high-resolution genotyping approach was applied to *P. falciparum *infected samples from Papua New Guinea (PNG) and Tanzania (TZ). These study sites differ extensively in their transmission intensity with entomological inoculation rates of >300 infective bites per person per year reported for TZ [14,15] and approximately 35 infective bites per person per year for PNG [16]. The main question was to assess whether the previously observed difference in *msp1 *and *msp2 *diversity in different endemic settings is independent of technical approaches or whether more sensitive detection of allelic size differences would alter these previous results. For direct standardized comparison the same genotyping technique was applied for all samples.

## Methods

The *msp1 *and *msp2 *loci were subject to high-resolution genotyping in two sets of DNA samples that had been PCR positive in previous tests. 108 samples from PNG were derived from a longitudinal field survey in one- to four-year old children, conducted in an area near Maprik, East Sepik Province (Lin *et al*, personal communication). Scientific approval and ethical clearance for the study was obtained from the Medical Research and Advisory Committee (MRAC) of the Ministry of Health in PNG and from the Ethikkommission beider Basel in Switzerland. Informed consent was sought from all parents or guardians prior to recruitment of each child. 115 Tanzanian samples were derived from the placebo group of a vaccine efficacy trial conducted from 1993-1994 in children below five years of age in the village Idete, which is located in the Kilombero District of Morogoro Region, Tanzania [17]. A genotyping study performed in these trial participants has been published previously [18], where the same marker gene *msp2*, but a different typing technique had been used. Both sets of samples derive from studies where morbid episodes were closely followed and anti-malarial treatment was readily available. In both sites, newly introduced drugs were administered, without evidence for existing drug resistance in these areas.

Prior to PCR amplification of the *msp1 *and *msp2 *target sequence, DNA was extracted from cell pellets using QIAamp^® ^96 DNA Blood Kits (Qiagen, Australia) according to the manufacturer's instructions. *Msp2 *genotyping was performed as previously described by Falk *et al *[1], with some minor changes and adaptations of PCR conditions for highly purified DNA. In brief, primary PCR reaction conditions were adjusted to a final volume of 50 μl including 2 μl of extracted DNA. Cycle conditions for primary PCR were 2 min at 94°C followed by 25 cycles of 30 s at 94°C, 45 s at 45°C, 90 s at 70°C, and a final extension at 70°C for 10 min. In order to reduce the carry over of primary PCR primers into the nested PCR, only one μl of primary PCR product was amplified in the nested PCR reaction with the following cycle conditions: 2 min at 94°C followed by 25 cycles of 30 s at 94°C, 45 s at 50°C, 90 s at 70°C and a final extension at 70°C for 10 min.

*Msp1 *occurs as one of three distinct allelic families: K1, MAD20 and RO33. The unique family specific sequences K1 and MAD20 flank intragenic repeat units that give rise to extensive size polymorphisms, whereas RO33 is not polymorphic. For amplification of the polymorphic region of *msp1 *block 2 [19], a nested PCR approach was used. Primary PCR was performed in a total volume of 50 μl containing 5 μl of 10 × BufferB (0.8 M Tris-HCl, 0.2 M (NH_4_)_2_SO_4_, 0.2% w/v Tween-20), 2 mM MgCl_2_, 200 μM dNTPs, 2.5 U Taq DNA polymerase (FirePol, Solis BioDyne). Primary PCR primers which are located in the conserved sequence spanning the *msp1 *block2 (M1-OF 5'-CTAGAAGCTTTAGAAGATGCAGTATTG-3' and M1-OR 5'-CTTAAATAGATTCTAATTCAAGTGGATCA-3' [20]) were used at a final concentration of 300 nM each. Two μl of DNA was used as template for this PCR reaction. An initial denaturation step of 94°C for 2 min was followed by 30 amplification cycles of 30 s at 94°C, 1 min at 54°C, 1 min at 72°C and a final extension for 5 min at 72°C.

In the nested PCR reaction, specific primer pairs were used to amplify the allelic families K1, MAD20 and RO33 of *msp1 *block2. In order to distinguish the size of PCR products by capillary electrophoresis, one of the primers for each PCR was labelled with the fluorescent dyes VIC, NED or 6-FAM, respectively (Applied Biosystems). Size variations due to sporadic addition of adenine by the Taq polymerase at the 3' end were avoided by adding a 7 bp tail (Applied Biosystems) to the 5' end of the other primer promoting the additional adenine incorporation. Primer sequences for nPCR have previously been published [20], but have been modified by fluorescent dyes and 7 bp tails: M1-KF 5'-Tail-AAATGAAGAAGAAATTACTACAAAAGGTGC-3'; M1-KR 5'-NED-GCTTGCATCAGCTGGAGGGCTTGCACCAGA-3'; M1-MF 5'-Tail-AAATGAAGGAACAAGTGGAACAGCTGTTAC-3'; M1-MR 5'-6-FAM-ATCTGAAGGATTTGTACGTCTTGAATTACC-3'. K1 and MAD20 allelic sequences were amplified in a duplex nPCR in a total volume of 50 μl, containing a final primer concentration of 100 nM for each primer, 5 μl of 10 × BufferB (0.8 mM Tris-HCl, 0.2 M (NH_4_)_2_SO_4_, 0.2% w/v Tween-29), 2 mM MgCl_2_, 200 μM dNTPs and 1.5 U Taq DNA polymerase (FirePol, Solis BioDyne). 1 μl of primary PCR product was used as template for the nested PCR with the following conditions: initial denaturation for 2 min at 94°C followed by 35 cycles of 30 s at 94°C, 1 min at 59°C, 1 min at 72°C and a final extension for 10 min at 72°C. RO33 allelic sequences were amplified with primers M1-RF 5' VIC-TAAAGGATGGAGCAAATACTCAAGTTGTTG-3' and the reverse primer M1-R2 5' Tail-CAAGTAATTTTGAACTCTATGTTTTAAATCAGCGTA-3' which is located in the conserved region of *msp1 *block3 and is not family specific. Therefore, RO33 nested PCR was run as a separate reaction under the same conditions as described for K1 and MAD20 nested PCR. All amplifications were performed on a PTC-100 thermocycler (MJ Research Inc.).

Nested PCR products were analysed on a 1.5% agarose gel. Depending on the intensity of the band, PCR products were diluted 1:5 - 1:40 in H_2_O. 2.5 μl of diluted PCR product was mixed with 0.3 μl GeneScan^®^-500 LIZ^® ^size standard (Applied Biosystems) and 12 μl HiDi (highly deionized) formamide. The mixture was heated for 5 min at 95°C to separate the double strands and then immediately chilled on ice for a few minutes before capillary electrophoresis was performed on an AB3130 Sequencer (Applied Biosystems).

Data were analysed using the GeneMapper^® ^v3.7 Software (Applied Biosystems). The results of size calling were exported as a tab delimited file and imported into an in-house generated software, which calculated for each sample a cut-off based on the mean height of the size standard peaks and grouped all alleles into 3 bp bins. For the cut-off, an empirically defined constant factor was multiplied by the mean size standard for each sample. This constant factor was manually defined after inspecting the background level in single infections. For both marker genes the total number of alleles as well as their frequency distributions were analysed. The theoretical probability of being infected by two parasites with the same allele was calculated as P = Σp_i_^2 ^where p_i _is the frequency of allele i [21]. The combined probability that two independent clones share the same genotype for both marker genes was calculated by multiplying the probabilities P for both marker genes, assuming that the two loci sort independently from each other. As a measure for genetic diversity, the expected heterozygosity was calculated by use of the formula H_E _= [n/(n-1)] × [(1-Σp_i_^2^)] [22], where n is the number of samples and p_i _the frequency of allele i. H_E _is the probability that two alleles randomly drawn from the population sample are different. The mean multiplicity of infection (MOI) was calculated as the total number of clones divided by the number of positive samples for each marker gene. Allele frequencies were further compared between populations from PNG and TZ using Wright's F statistics to calculate the fixation index F_ST_. F_ST _is a measure of between population variance and gives the proportion of overall diversity which is attributable to differences between populations [23]. H_E _and F_ST _values were calculated by Arlequin ver3.1 software [24].

Estimates were calculated for both, the frequency distribution and the mean MOI adjusted for the probability of multiple infections sharing the same allele using the *msp2 *allele frequency distribution and the distribution of observed numbers of infections according to the method of Ross *et al *(personal communication).

## Results and discussion

**Figure 1:**
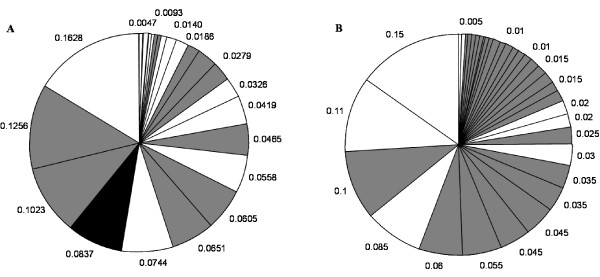
**Allelic frequencies of *msp1 *and *msp2 *in samples from Papua New Guinea**. **A**. White, grey and black areas indicate frequencies of K1, MAD20 and RO33 alleles, respectively. **B**. White and grey areas indicate Fc27 and 3D7 allele frequencies, respectively.

**Figure 2:**
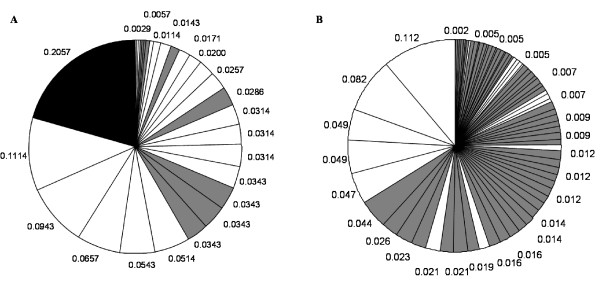
**Allelic frequencies of *msp1 *and *msp2 *in samples from Tanzania**. **A**. White, grey and black areas indicate frequencies of K1, MAD20 and RO33 alleles, respectively. **B**. White and grey areas indicate Fc27 and 3D7 allele frequencies, respectively.

The distribution of *msp1 *and *msp2 *allele frequencies for 108 Papua New Guinean samples is shown in Figure 1. The total number of alleles detected in this sample set is greater for *msp2 *than *msp1*, with 35 vs. 24 differently sized alleles, respectively. The overall genetic diversity is also slightly greater for *msp2 *than *msp1 *(P = 0.07; H_E _= 0.933 for *msp2 *and P = 0.084; H_E _= 0.918 for *msp1*). By combining the two marker genes, the probability that two parasite clones share the same genotype by chance can be reduced to 0.0058. Figure 2 shows the allelic frequency distribution of *msp1 *and *msp2 *in 115 samples from Tanzania. In the Tanzanian samples, the total number of *msp2 *alleles detected by CE was much greater than for *msp1 *with 76 vs. 29 alleles discriminated, respectively. The probability P of being infected by two parasites with the same allele and the expected heterozygosity values H_E _showed a greater overall diversity for *msp2 *compared to *msp1 *(P = 0.036; H_E _= 0.965 for *msp2 *and P = 0.085; H_E _= 0.917 for *msp1*) in Tanzanian samples. Combining the two marker genes reduced the probability that two parasites share the same genotype by chance to 0.003.

In both countries, *msp1 *K1 type alleles were the most polymorphic, followed by MAD20 type alleles, whereas RO33 was non-polymorphic. For *msp2*, alleles belonging to the 3D7 family showed much more polymorphism than Fc27 type alleles, in both countries (Table 1). For *msp1*, the most dominant alleles were similar between PNG and TZ. For *msp2 *the most frequent allele was the same between the two sites whereas the remaining alleles showed a different order of frequency. It is clear that measuring length polymorphism only detects a fraction of diversity in the marker genes. However, for genotyping multiple infections, extensive length polymorphism is ideal for discriminating individual infections. Sequencing would require cloning individual PCR fragments. The variance in allele frequencies between the two study sites was compared using Wright's F statistics. The F_ST _values for pair wise comparisons of allele frequencies between population samples were 0.041 for *msp1 *and 0.017 for *msp2*, respectively (p < 0.001 for both markers). These low F_ST _values indicate that allele frequencies are highly similar and that there is little genetic differentiation between the investigated geographic populations for both marker genes. As previously suggested, such low F_ST _values may indicate that the *msp1 *and *msp2 *loci are under balancing selection [25], increasing the possibility that observed patterns of allele frequencies are the result of balancing selection rather than of variation in transmission dynamics.

**Table 1:** Number of different *msp1 *and *msp2 *alleles detected in samples from Papua New Guinea and Tanzania

	**Papua New Guinea**	**Tanzania**
Number of different *msp1*-K1 alleles	13	19

Number of different *msp1*-MAD20 alleles	10	9

Number of different *msp1*-RO33 alleles	1	1

Number of different *msp2*-3D7 alleles	27	59

Number of different *msp2*-Fc27 alleles	8	17

The quality of genotyping, i.e. optimal differentiation of parasite clones within a blood sample and between samples depends on a number of parameters. It is essential that the chosen marker gene is highly polymorphic. Many genotyping applications require minimization of the probability that two parasites share the same allele by chance. Minimizing this probability is achieved by choosing the most polymorphic marker, but also the allelic frequency distribution plays a crucial role and should be homogeneous. The more homogeneous the distribution of alleles, the more diversity the marker will have [21].

Discrimination can be enhanced by using a second marker gene, which further reduces the probability that two clones share the same genotype by chance. In fact, the number of marker genes that should be used to adequately discriminate parasites can be different between study sites and it has been recommended to choose the number of genes in a way that this probability is below 0.05 [21]. The study presented here has shown that in Tanzania, the *msp2 *gene is highly diverse with a total of 76 differently sized alleles, and that the probability P of being infected with two parasites sharing the same *msp2 *genotype is only 3.6%. In contrast, the frequency distribution of the *msp1 *gene in Tanzania is less favourable, since only 29 alleles could be distinguished and the most common allele has a frequency of >20%. These results suggest that in a high transmission region in Tanzania, where this study was conducted, CE genotyping for *msp2 *alone provided sufficient discrimination power to adequately differentiate parasites, since the probability P that two parasites share the same genotype was 3.6%. For *msp1 *however, the genetic diversity was not sufficient for use as a single genotyping marker. The number of alleles was greater for both marker genes in Tanzanian samples than in samples from Papua New Guinea (*msp1*: 24 vs. 29; *msp2*: 35 vs. 76 in PNG vs. TZ, respectively, Table 2). Although the overall genetic diversity in PNG was slightly greater for *msp2 *than for *msp1 *with 35 vs. 24 alleles, the diversity of *msp2 *in this area was not high enough for *msp2 *to be used as a single marker for genotyping. This is due to a high probability (7%) of being infected with two parasites sharing the same allele by chance, which is above the 5% threshold suggested by Gatton & Cheng [21]. Therefore, genotyping of both *msp2 *and *msp1 *is required in order to increase discriminatory power in this area. Combining the two marker genes reduced the probability that two parasites share the same genotype by chance to 0.6%. In an area of lower transmission, genotyping two or more markers is clearly an option for single clone infections as *msp1-msp2 *haplotypes are evident. In the case of frequent multiple clone infections, this strategy is less beneficial as both markers are unlinked and *msp1-msp2 *haplotypes cannot be determined. This shortfall poses a serious handicap if an individual parasite clone needs to be followed up over time in a longitudinal series of samples. Other applications, e.g. identification of new infections, are well suited for combining several markers.

**Table 2:** Diversity of *msp1 *and *msp2 *in samples from Papua New Guinea and Tanzania

	**Papua New Guinea**		**Tanzania**	
	
	***msp2***	***msp1***	***msp2***	***msp1***
Number of samples (n)	108	108	115	115

Number of alleles	35	24	76	29

Frequency of most common allele	15.08%	16.28%	11.21%	20.57%

Number of clones	199	215	428	350

Mean MOI*	1.84	1.99	3.72^§^	3.04^§^

H_E_**	0.933	0.918	0.965	0.917

P = Σp_i_^2^	0.07	0.084	0.036	0.085

Combined probability***	0.0058		0.003	

* MOI = mean multiplicity of infection

** H_E _= expected heterozygosity. This is defined as the probability that two randomly chosen alleles are different in the population.

*** The combined probability that two independent clones share the same genotype for both marker genes was calculated by multiplying the probabilities P for both marker genes.

^§ ^Indicates significant difference between mean MOI of *msp1 *and *msp2 *(p < 0.001)

Mean MOI was significantly greater in Tanzania than in PNG for both marker genes (*msp1*: 1.99 vs. 3.04; p < 0.001 and *msp2*: 1.84 vs. 3.72, p < 0.001 for PNG vs. TZ, respectively, Table 2), which confirms previous observations of an increased complexity of infection with increasing endemicity [3,26]. In Tanzanian samples, the mean MOI was significantly greater for *msp2 *than for *msp1 *(p < 0.001), which reflects the great difference in the number of distinct alleles between the two allelic families. Despite the fact that the number of alleles was also greater for *msp2 *than for *msp1 *in PNG, the mean multiplicity of infection was slightly higher for *msp1*, however, this difference was not statistically significant. This minor difference in mean MOI might be due to various technical reasons like differences in PCR efficiency for the two marker genes, which results in different detection limits. Another parameter that influences the number of genotypes per sample is the cut-off that is determined for each sample, which is dependent on the internal size standard used to control for unequal loading of the PCR product onto the automated sequencer. The use of two markers obviously produces minor discrepancies due to technical differences, but does not affect the overall MOI result.

Because MOI was considerably higher in samples from TZ than in those from PNG, the total number of clones used for assessing diversity differed between the two data sets. This could have biased the results in that the diversity in PNG would be under-estimated by observing fewer clones. Therefore, the analysis of the *msp2 *diversity was repeated with a two-fold increased number of samples from PNG, so that the total number of clones was approximately equal in both data sets (data shown in additional files 1 and 2). A minor increase in the number of distinct alleles was observed, with four newly observed alleles occurring only 1-3 times. Thus, comparing approximately 400 clones from each site did not yield a different estimate of diversity as opposed to comparing approximately 100 blood samples form each site. The expected heterozygosity H_E _and the probability P that two parasites share the same allele by chance remained similar.

The results obtained by applying this high-resolution genotyping technique revealed similar results to previous reports on allelic diversities in three areas of different malaria endemicity in Brazil, Vietnam and Tanzania, where the extent of allelic diversity of *msp2 *as estimated by the total number of distinct alleles increased with increasing endemicity [4]. The same populations were also investigated for genetic diversity in the *msp1 *gene. In contrast to *msp2*, the extent of *msp1 *diversity did not seem to correlate with the level of malaria transmission in these regions [5-9]. The present observations of only a minor effect of transmission intensity on diversity of *msp1 *confirmed these previous findings. An increased diversity of *msp2 *in areas of higher endemicity was also reported previously [3,10,11,27], however, most of these studies also reported a correlation between endemicity and the number of distinct alleles for *msp1*, which was not the case for the comparison of diversity between Brazil, Vietnam and Tanzania and in the present study. There were also some studies that did not find a correlation between transmission intensity and genetic diversity for both marker genes [12,28,29].

The findings presented here might have implications for genotyping samples from drug efficacy trials where recrudescence must be reliably distinguished from new infections and the probability of new infecting parasites having the same allele as the initial infecting parasite should be as low as possible. The data presented here suggest that for this purpose *msp2 *is the more suitable marker gene than *msp1 *in both study sites. Genotyping only *msp1 *would not provide adequate discriminatory power according to the standards suggested by Gatton and Cheng [21] as the probability of being reinfected with the same genotype is 8.5%. In areas of lower transmission intensity, such as in PNG, genotyping only one marker gene (either *msp1 *or *msp2*) will not provide enough discriminatory power and two markers are necessary to improve discrimination power. In highly endemic areas like Tanzania, the resolution obtained with *msp2 *might be sufficient to discriminate all concurrent clones within an individual, as the probability of multiple parasites sharing the same allele is low. However, the high mean MOI in this area is likely to lower the discriminatory power. This effect of high MOI on the resolution of genotyping markers has so far been given little attention [13] in genotyping studies. It is of relevance in particular for genotyping of recrudescences in highly endemic areas, because high MOI increases the probability of super-infected parasite clones to carry the same genotype. Therefore an attempt was made to estimate the true number of alleles present in a host, allowing for this probability. Details of the estimation approach are being published elsewhere (Ross *et al*, personal communication). Table 3 lists the probability of observed infections conditional on the true number of infections based on the PNG allelic frequencies. Adjustment for the probability of multiple infections sharing the same *msp2 *allele made little difference to the estimated MOI (estimated mean MOI was 1.84 in PNG, 3.99 in Tanzania compared to the unadjusted values of 1.84 in PNG and 3.72 in TZ, respectively). This reflects the low probability of being infected with two parasites sharing the same allele with such a high-resolution typing system.

**Table 3:** Probability of each number of observed infections, conditional on the true number of infections

	**1**	**2**	**3**	**4**	**5**
1	1	0.068	0.007	0.0008	9.38E-05
2		0.931	0.185	0.0358	0.007077
3			0.808	0.3108	0.097212
4				0.6526	0.404946
5					0.490671

Rows indicate the number of observed infections, whereas columns represent the true number of infections. Results are shown for MOI ≤ 5.

## Conclusion

In conclusion, the CE-based genotyping, which provides highly accurate fragment sizing data, is in line with previous findings on genetic diversity in different geographic locations. A higher mean MOI for both marker genes was found in an area of more intense malaria transmission. *Msp2 *diversity was higher in the high transmission area, but *msp1 *only showed a minor increase in diversity. The between population variance in allele frequencies, as estimated by Wright's F statistics, was found to be very low for both marker genes. This indicates that there was little genetic differentiation between the two sites of different endemicity and suggests that the observed patterns of allele frequencies are independent of transmission intensity.

## Competing interests

The authors declare that they have no competing interests.

## Authors' contributions

SS carried out the molecular genetic work and data analysis. FV participated in the molecular genetic work. EL and BK carried out the field work in PNG. IM was responsible for the field study in PNG and assisted in designing the study. IF was responsible for the study design and contributed to data analysis. All authors contributed to writing the manuscript.

## Supplementary Material

Additional file 1 **Diversity of *msp2 *in samples from Papua New Guinea.** Click here for file

Additional file 2 **Allelic frequencies of *msp2 *in samples from Papua New Guinea**. White and grey areas indicate Fc27 and 3D7 allele frequencies, respectively. Click here for file
