# Supplementary material for: Comparison of Plasmodium falciparum allelic frequency distribution in different endemic settings by high-resolution genotyping
Source: Malar J. 2009 Oct 30;8:250. doi: 10.1186/1475-2875-8-250 (PMC2774868; doi:10.1186/1475-2875-8-250)
Supplement: Additional file 1 — Diversity of msp2 in samples from Papua New Guinea. [file 1475-2875-8-250-S1.doc]

**Supplementary material**

Additional file 1: Diversity of *msp2* in samples from Papua New Guinea

|  | Papua New Guinea |
| --- | --- |
|  | *msp2* |
| Number of samples (n) | 217 |
| Number of alleles | 39 |
| Frequency of most common allele | 15.4% |
| Number of clones | 369 |
| Mean MOI^*^ | 1.7 |
| H_E_^**^ | 0.933 |
| P=∑p_i_^2^ | 0.07 |

^*^ MOI = mean multiplicity of infection

^**^ H_E_ = expected heterozygosity. This is defined as the probability that two randomly chosen alleles are different in the population.
