# Supplementary figures and images for: Comparison of Plasmodium falciparum allelic frequency distribution in different endemic settings by high-resolution genotyping
Source: Malar J. 2009 Oct 30;8:250. doi: 10.1186/1475-2875-8-250 (PMC2774868; doi:10.1186/1475-2875-8-250)

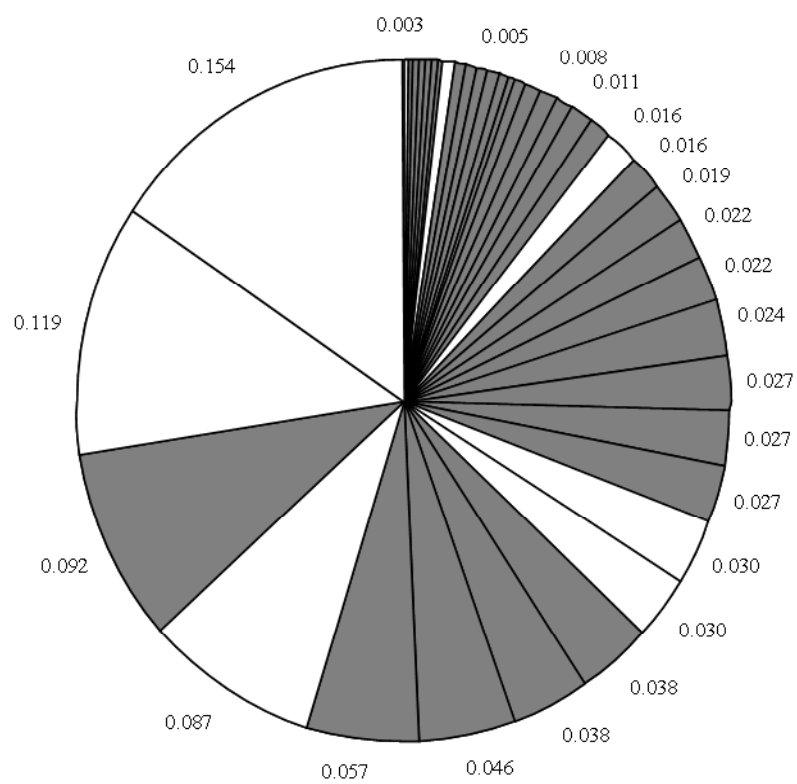

Supplement: Additional file 2 — Allelic frequencies of msp2 in samples from Papua New Guinea. White and grey areas indicate Fc27 and 3D7 allele frequencies, respectively. [file 1475-2875-8-250-S2.pdf]
